# Detrimental Effects of Non-Functional Spermatozoa on the Freezability of Functional Spermatozoa from Boar Ejaculate

**DOI:** 10.1371/journal.pone.0036550

**Published:** 2012-05-02

**Authors:** Maria J. Martinez-Alborcia, Anthony Valverde, Inmaculada Parrilla, Juan M. Vazquez, Emilio A. Martinez, Jordi Roca

**Affiliations:** 1 Department of Medicine and Animal Surgery, Faculty of Veterinary Science. University of Murcia, Murcia, Spain; 2 Agronomy School, Costa Rica Institute of Technology, Cartago, Costa Rica; University Hospital of Münster, Germany

## Abstract

In the present study, the impact of non-functional spermatozoa on the cryopreservation success of functional boar spermatozoa was evaluated. Fifteen sperm-rich ejaculate fractions collected from five fertile boars were frozen with different proportions of induced non-functional sperm (0 –native semen sample-, 25, 50 and 75% non-functional spermatozoa). After thawing, the recovery of motile and viable spermatozoa was assessed, and the functional of the spermatozoa was evaluated from plasma membrane fluidity and intracellular reactive oxygen species (ROS) generation upon exposure to capacitation conditions. In addition, the lipid peroxidation of the plasma membrane was assessed by the indirect measurement of malondialdehyde (MDA) generation. The normalized (with respect to a native semen sample) sperm motility (assessed by CASA) and viability (cytometrically assessed after staining with Hoechst 33342, propidium iodide and fluorescein-conjugated peanut agglutinin) decreased (p<0.01) as the proportion of functional spermatozoa in the semen samples before freezing decreased, irrespective of the semen donor. However, the magnitude of the effect differed (p<0.01) among boars. Moreover, semen samples with the largest non-functional sperm subpopulation before freezing showed the highest (p<0.01) levels of MDA after thawing. The thawed viable spermatozoa of semen samples with a high proportion of non-functional spermatozoa before freezing were also functionally different from those of samples with a low proportion of non-functional spermatozoa. These differences consisted of higher (p<0.01) levels of intracellular ROS generation (assessed with 5-(and-6) chloromethyl-20,70-dichlorodihydrofluorescein diacetate acetyl ester; CM-H_2_DCFDA) and increased (p<0.01) membrane fluidity (assessed with Merocyanine 540). These findings indicate that non-functional spermatozoa in the semen samples before freezing negatively influence the freezability of functional spermatozoa.

## Introduction

Frozen-thawed (FT) spermatozoa have advantages over liquid-stored semen for swine industry [1]. However, they are still rarely used in swine commercial artificial insemination (AI) programs because of their lower efficiency with respect to liquid-stored semen [2], [3], i.e., more FT spermatozoa are required per AI dose to achieve usually lower fertility outcomes [4]. The biological reason for the large number of spermatozoa required per AI dose is related to the high susceptibility of boar spermatozoa to damage from the cryopreservation process; cryosurvival rates below 50–60% are typical [5]. In addition, a population of cryosurvived spermatozoa has altered functionality [6]. Therefore, the optimization of cryopreservation protocols is of primary importance for the efficient application of FT semen in the swine industry.

In addition to the particular sensitivity of boar spermatozoa to cooling, which is caused by a peculiar membrane lipid composition [7], other factors, such as the individual boar's physiology and semen traits as well as the semen handling during cryopreservation, contribute to the poor sperm freezability [5], [8], [9]. In relation to the semen traits, boar ejaculates contain spermatozoa with morphological or structural alterations that comprise a subpopulation of non-functional spermatozoa whose numbers vary among boars and among ejaculates within each boar. Large proportions of non-functional sperm in the ejaculates of mammalian species are associated with impairment of oocyte fertility, poor embryo quality and suboptimal pregnancy rates [10]. Although the relationship between semen traits and sperm quality after thawing is still controversial [5], [11], it is a common practice to select only those ejaculates with a low proportion of non-functional spermatozoa for cryopreservation. This selection is performed to improve the efficiency of the cryopreservation process; more functional spermatozoa can be recovered after thawing because more functional spermatozoa were in the semen samples before freezing, which allows generation of more insemination doses. However, to the best of our knowledge, no specific studies have evaluated whether non-functional spermatozoa present in the semen samples before freezing influence the freezability of the functional spermatozoa. As has been described in other species, such as the bovine [12], non-functional spermatozoa present in the semen samples are a source of reactive oxygen species (ROS), which have a detrimental effect on the functional sperm population. It is therefore likely that the non-functional sperm subpopulation negatively influences the freezability of the functional sperm subpopulation. Boar spermatozoa are particularly sensitive to lipid peroxidation because of the high content of unsaturated fatty acids in the plasma membrane [4].

Current boar sperm cryopreservation protocols include the storage of the ejaculate at a temperature greater than 15°C for several hours before freezing [4], which facilitates the shipping from the site of collection to the freezing facility [13]. Although this long storage period before freezing, during which the ejaculated spermatozoa remain in contact with their own seminal plasma (SP), improves sperm freezability [14], [15], it also allows a lengthy contact period of non-functional with functional spermatozoa.

The present study aimed to evaluate the impact of non-functional sperm subpopulations in the semen sample on the ability of functional spermatozoa to withstand the cryopreservation process. We developed an experimental approach in which semen samples with different proportions of induced non-functional sperm subpopulation were cryopreserved. The impact of non-functional spermatozoa on the cryopreservation success of functional spermatozoa was evaluated after thawing by assessing the recovery of motile, viable and functional spermatozoa and evaluating the dynamic changes experienced by thawed spermatozoa exposed to capacitation conditions.

## Materials and Methods

### Reagents and media

All chemicals used in the experiments were of analytical grade. Unless stated otherwise, all media components were purchased from Sigma-Aldrich (St. Louis, MO, USA), and the media were prepared under sterile conditions in a laminar flow hood (MicroH; Telstar, Terrasa, Spain).

The basic medium used to dilute the spermatozoa was Beltsville Thawing Solution (BTS: 205 mM glucose; 20.4 mM sodium citrate; 5.4 mM KCl; 15.0 mM NaHCO_3_; 3.35 mM EDTA; pH 7.2; 321±3 mosmol/kg) [16] supplemented with 50 µg/ml kanamycin sulfate. Phosphate-buffered saline (PBS: 137 mM NaCl; 2.7 mM KCl; 0.86 mM NaH_2_PO_4_; 6.4 mM Na_2_HPO_4_·7H_2_O; pH 6.8; 289±4 mosmol/kg) [17] was used to dilute the sperm samples for flow cytometric analysis. The spermatozoa were frozen in basic freezing medium (FE: 80% (v/v) Tris-citric acid-glucose extender (111 mM Trizma Base; 31.4 mM monohydrate citric acid, 185 mM glucose), 20% (v/v) egg yolk; 100 µg/ml kanamycin sulfate; pH 7.2; 299±5 mosmol/kg) [5].

The sperm samples were incubated under capacitating conditions to evaluate the fluidity of the plasma membrane and the intracellular ROS production of thawed spermatozoa. The capacitation medium was Tris-buffered basal medium (20 mM Tris; 131.1 mM NaCl; 3 mM KCl; 11 mM glucose; 5 mM sodium pyruvate and 10 µg/ml phenol red; at pH 7.4 and 299±2 mosmol/kg) supplemented with 7.5 mM CaCl_2_·2H_2_O, 1 mM caffeine and 0.2 mg/ml bovine serum albumin fraction V at pH 7.4 and 300±2 mosmol/kg (mTBM); and equilibrated for 48 h in the dark at 39°C under an atmosphere of 5% (v/v) CO_2_ and 100% (v/v) humidity [17].

### Collection, evaluation and processing of the ejaculates

All procedures that involved animals were performed according to international guidelines and approved by the Bioethics Committee of Murcia University (research code: 331/2008).

All ejaculates were collected from healthy and sexually mature boars (2–4 years of age) that were fertile and undergoing regular semen collection for commercial AI. None of the boars were preselected according to sperm freezability. The boars were housed in individual pens in an environmentally controlled (15–25°C) building with windows so that the animals were exposed to natural daylight and supplementary light for a total of 16 h of light per day. The boars had free access to water and were fed a commercial diet according to the nutritional requirements for adult boars [18].

Sperm-rich ejaculate fractions (SREFs) were collected using the gloved-hand method. The ejaculates were then extended in BTS (1∶1, v/v) and evaluated for conventional semen characteristics. Only ejaculates containing more than 200×10^6^ sperm/ml (evaluated in a colorimeter), 70% motile spermatozoa (subjectively evaluated by light microscopy), 80% spermatozoa with normal morphology and 80% spermatozoa with intact acrosome ridges (evaluated with phase contrast microscopy of sperm samples fixed in buffered 2% (v/v) glutaraldehyde) were selected. After the evaluation, the diluted SREFs were dispensed into 50 ml plastic tubes, slowly cooled to 17–18°C, packaged in insulated containers and shipped to the Andrology Laboratory at the Veterinary Teaching Hospital (VTH) of the University of Murcia with temperature monitoring by a miniature data logger (Gemini Data Loggers, Ltd., Chichester, UK). The diluted SREFs arrived at the laboratory within 2 h of semen collection.

### Sperm cryopreservation

The semen samples were centrifuged (Megafuge 1.0 R, Heraeus, Hanau, Germany) for 3 min at 2,400 *g*, and the sperm pellets were frozen by using the straw freezing procedure described by Hernandez et al [9]. Briefly, sperm pellets were diluted in FE to 1.5×10^9^ cells/ml. After cooling to 5°C for 120 min, the extended sperm cells were diluted with FE-glycerol-Equex extender (89.5% FE+1.5% Equex STM (v/v) (Nova Chemical Sales, Scituate, MA, USA) and 9% glycerol (v/v); pH 6.2; 1,715±15 mosmol/kg) to a final concentration of 1×10^9^ cells/ml. The spermatozoa were then packed into 0.5 ml polyvinyl chloride (PVC) French straws (Minitüb, Tiefenbach, Germany) and frozen using a controlled-rate freezing machine (IceCube 1810: Minitüb) to −5°C at a rate of 6°C/min and from −5°C to −80°C at a rate of 40°C/min, held for 30 s at −80°C, further cooled to −150°C at a rate of 70°C/min and then plunged into liquid nitrogen for storage at −196°C. The straws remained in liquid nitrogen for at least 1 week before thawing, which was performed in a circulating water bath at 37°C for 20 s. The thawed semen samples were diluted in BTS (1∶1 v/v) and incubated at 37°C for 30 min, when sperm quality and functionality were assessed.

### Sperm assessments

The spermatozoa were assessed according to conventional quality parameters (motility and viability) and functionality. Functional was evaluated in terms of plasma membrane fluidity and intracellular ROS production. All these assessments, except motility, which was evaluated using a computer-assisted analysis, were performed by flow cytometry. The membrane lipid peroxidation (LPO) was also evaluated by the indirect measurement of the generation of malondialdehyde (MDA).

The flow cytometric analyses were performed at room temperature under dimmed light in a BD FACSCanto II flow cytometer (Becton Dickinson & Company, Franklin Lakes, NJ, USA) equipped with three lasers as excitation sources: blue (488 nm, air cooled, 20 mW solid state), red (633 nm, 17 mW HeNe), and violet (405 nm, 30 mW solid state). The data were acquired using BD FACSDiva Software (Becton Dickinson). The non-sperm events were gated out on the basis of Hoechst 33342 (H-42) fluorescence (DNA content), and the acquisitions were stopped after 10,000 H-42–positive events were recorded. The fluorescence spectrum of H-42 was detected using a 450/50 nm band-pass (BP) filter.

### Sperm motility and viability

The motility of the spermatozoa was evaluated objectively by using a computer-assisted analysis system (ISAS; Proiser R+D, Paterna, Spain) as described by Cremades et al [19]. Briefly, the sperm samples were suspended in BTS to a concentration of 25–30×10^6^ cells/ml. For each evaluation, a 5 µl aliquot of each sperm sample was placed in a Makler counting chamber (Sefi Medical Instruments, Haifa, Israel) that was pre-warmed to a 38°C, and six to nine fields were visualized to analyze a minimum of 600 spermatozoa per sample. Before the track sequence was analyzed, the trajectory of each spermatozoon identified and recorded in each field was assessed visually to eliminate possible debris and to decrease the risk of including unclear tracks in the analysis. The sperm motility variable recorded was the overall percentage of motile spermatozoa (average path velocity (VAP)> = 20 µm/s).

The viability of the spermatozoa was evaluated by simultaneous cytometric assessment of the plasma and acrosomal membrane integrity by using a triple-fluorescence procedure. Briefly, a 100 µl sperm sample (30×10^6^ cells/ml in PBS) was transferred to culture tubes containing 2.5 µl H-42 (0.05 mg/ml in PBS), 2 µl propidium iodide (PI, 0.5 mg/ml in PBS, Molecular Probes Europe BV, Leiden, The Netherlands) and 2 µl fluorescein-conjugated peanut agglutinin (PNA-FITC, 200 µg/ml in PBS). The samples were mixed and incubated at 38°C in the dark for 10 min. Immediately before analysis by flow cytometry, 400 µl PBS was added to each sample, and the samples were mixed. The fluorescence spectra of PI and PNA-FITC were detected by use of a 670 nm long-pass (LP) filter and a 530/30 nm BP filter, respectively. The spermatozoa analyzed were categorized into four categories: (1) intact plasma and acrosomal membranes (PI^−^/PNA^−^FITC^−^); (2) intact plasma membrane and damaged acrosome (PI^−^/PNA^−^FITC^+^); (3) damaged plasma membrane and intact acrosome (PI^+^/PNA^−^FITC^−^); or (4) damaged plasma and acrosomal membranes (PI^+^/PNA^+^). The viable spermatozoa exhibited intact plasma and acrosomal membranes and this was expressed as a percentage of the total cells.

### Sperm functional

The functionality of the spermatozoa was assessed by the plasma membrane fluidity and intracellular ROS production in sperm samples incubated under capacitating conditions; the samples were diluted in mTBM at a concentration of 30×10^6^ cells/ml and incubated for 15 min in the dark at 39°C under an atmosphere of 5% (v/v) CO_2_ and 100% (v/v) humidity [17].

The fluidity of the plasma membranes was assessed by staining with H-42, Merocyanine 540 (M-540) and Yo-Pro-1 (Molecular Probes Europe BV, Leiden, The Netherlands). Merocyanine 540 detects changes in membrane fluidity during sperm capacitation [20]. Aliquots of 50 µl of mTBM-diluted spermatozoa were diluted in 950 µl mTBM containing 1.25 µl H-42 (0.05 mg/ml in PBS) and 1 µl Yo-Pro-1 (25 µM in DMSO) and incubated at 38°C in the dark for 8 min under an atmosphere of 5% (v/v) CO_2_ and 100% (v/v) humidity. Then, 2.6 µl M-540 (1 mM in DMSO) was added to all samples, which were incubated for a further 2 min under the same conditions before flow-cytometric analysis. The fluorescence spectrum of M-540 was detected with a 670 nm LP filter, and Yo-Pro-1 was detected with a 530/30 nm BP filter. The spermatozoa were categorized as (1) viable cells with low plasma membrane fluidity (Yo-Pro-1^−^/M-540); (2) viable cells with high plasma membrane fluidity (Yo-Pro-1^−^/M-540^+^); or (3) non-viable cells (Yo-Pro-1^+^). Only the percentage of viable cells with high plasma membrane fluidity is reported.

Spontaneous intracellular ROS generation is common during sperm capacitation [21]. The intracellular production of ROS by sperm samples was measured in the present study by using 5-(and-6) chloromethyl-20,70-dichlorodihydrofluorescein diacetate acetyl ester (CM-H_2_DCFDA), which is freely permeable across cell membranes and becomes incorporated into the hydrophobic regions of the cell. Upon entering the cell, the acetate moiety of CM-H_2_DCFDA is cleaved by cellular esterases to leave the impermeant and non-fluorescent molecule 20,70-dichlorodihydrofluorescein (H_2_DCF). The H_2_DCF is oxidized by hydrogen peroxide (H_2_O_2_) into dichlorofluorescein (DCF), which fluoresces at 530 nm following excitation at 488 nm [22]. For each sperm sample, two different 50 µl aliquots of mTBM-diluted spermatozoa were diluted in 950 µl PBS containing (1) 1.25 µl H-42 (0.05 mg/ml in PBS), 1 µl PI (1 mg/ml in PBS), 1 µl H_2_DCFDA (1 mM in DMSO) and 1 µl tert-butylhydrogen peroxide (1 mM in purified water) to induce oxidative stress (first aliquot; used to measure induced ROS formation) or (2) 1.25 µl H-42, 1 µl PI and 1 µl H_2_DCFDA (second aliquot; used to measure basal ROS formation). The samples were incubated at 38°C in the dark for 10 min before flow cytometry. The mean fluorescence intensity of DCF (induced minus basal) was expressed as fluorescence units (FU) per 10^12^ live spermatozoa.

### Measurement of membrane lipid peroxidation

Lipid peroxidation is a sensitive indicator of oxidative stress in boar spermatozoa [23], and MDA is used as an indicator of lipid peroxidation in a variety of cell types, including spermatozoa [24]. The sperm membrane LPO was assessed via the indirect measurement of the generation of MDA by use of the BIOXYTECH MDA-586 Assay Kit (OxisResearch, Burlingame, CA, USA) and a modification of the procedure described by Gérard-Monnier et al [25]. This assay quantifies MDA using the reaction between *N*-methyl-2-phenylindole and MDA (45°C for 1 h in presence of FeSO_4_·7H_2_O), which yields a stable chromophore that can be measured at 586 nm. BTS-diluted sperm samples (3×10^6^ cells) were incubated for 30 min at 37°C in the presence of 1 µl of Fe^2+^/ascorbate solution (11 mg FeSO_4_·7H_2_O and 40 mg sodium ascorbate in 10 ml of distilled water) to promote the lipid peroxidation cascade and MDA release [26]. The incubated sperm sample (50 µl) was mixed with 2 µl of probucol, 160 µl of 1-methyl-2-phenylindole (diluted in acetonitrile/methanol [3∶1 v∶v]) and 37.5 µl of 12 N HCl; incubated at 45°C for 60 min; and centrifuged at 10,000 *g* for 10 min to pellet the precipitate. A 200 µl aliquot of the clear supernatant was collected and transferred to a 96-well flat-bottom transparent plate (Greiner Bio-One, Frickenhausen, Germany). A calibration curve prepared from a MDA stock (1,1,3,3-tetramethoxypropane) was included on the plate, and the absorbance at 586 nm was read in a spectrophotometer (PowerWave XS, Bio-Tek, Winooski, Vermont, USA). The LPO index was calculated as micromoles of MDA per 30×10^6^ spermatozoa. This assay was performed four times for each sample and the mean of each four was taken as the result.

### Experimental design

In this experiment, 15 diluted SREFs (BTS, 1∶1, v/v) from five hybrid boars (3 SREFs per boar) were processed. Upon arrival at the laboratory of Veterinary Teaching Hospital, the diluted SREFs were evaluated (initial semen traits) for sperm concentration (Nucleocunter-SP100, Chemometec, Allerød, Denmark), motility and viability. Thereafter, each extended SREF was divided into two aliquots: A and B. Aliquot A remained untreated (native semen sample). Aliquot B was used to generate the population of non-functional spermatozoa by freeze-thaw cycles in the absence of cryoprotectant for mimicking the damaging effects undergoing by those spermatozoa unable to sustain and adapt to the thermo-physical stresses associated to the freezing and thawing process. For this, 15 mL tubes of diluted SREF were immersed in liquid nitrogen for 9 min and immediately heated at 80°C in a water bath for 3 min, time at which the sample reached 35–36°C. This procedure was repeated three times to ensure that no spermatozoa remained viable, which was confirmed by the cytometric evaluation of sperm viability.

Four different semen samples were then generated by mixing aliquots A and B in different proportions. The first semen sample included only aliquot A (native semen sample). To generate the other three semen samples, aliquots A and B were mixed to obtain 25, 50 and 75% of non-functional spermatozoa. Thereafter, the resulting semen samples were stored at 17°C for 16 h before freezing. This long storage was performed because boar ejaculates are currently shipped overnight from the site of collection to the freezing facility [13]. Furthermore, the resistance of spermatozoa to cold injury increases when the pre-freezing storage time is increased to 16 h [14], [15].

The influence of non-functional spermatozoa on the cryopreservation success of functional spermatozoa was assessed at 30 min after thawing using three separate straws per semen sample. The percentages of motile and viable spermatozoa after thawing from each semen sample were normalized to those before freezing (initial semen traits) from the equation:
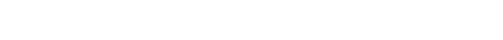



The number of thawed viable spermatozoa with high plasma membrane fluidity in each semen sample was normalized to the total number of thawed, viable spermatozoa counted in the same semen sample to obtain a percentage.

### Statistical analysis

Statistical analyses were performed using SPSS, version 15 software (SPSS Inc., Chicago, IL). The data were first evaluated using the Kolmogorov–Smirnov test to test for normality. Variables that were not normally distributed were either arcsine- (percentage data) or log-transformed (non-percentage data) before statistical analysis. The data were analyzed as a split-plot design by using a mixed linear ANOVA that included the fixed effects of boar and semen sample (100, 75, 50 and 25% functional spermatozoa) within each boar, with the random effect of replicate. Where appropriate, the Bonferroni test was used for post-hoc analyses. p<0.05 was considered to be statistically significant. All of the data are shown as the means ± SEMs.

## Results

### Initial semen traits and sperm quality after thawing

The mean values for semen parameters before freezing and the sperm quality variables assessed after the thawing of 15 ejaculates from the five boars used in this study are summarized in Table 1. All of the ejaculates had good sperm quantity and quality before freezing, with more than 200×10^6^ spermatozoa/ml, 90% normal sperm morphology, 70% motile spermatozoa and 75% viable spermatozoa. Of the semen traits evaluated, only sperm concentration (p<0.01) and viability (p<0.05) differed among boars. In contrast, differences (p<0.01) among boars were evident for all sperm parameters after thawing of the native semen samples. Boars 1 and 2 exhibited the best and the worst sperm quality, respectively. In all boars, the sperm quality after thawing differed (p<0.01) from that observed before freezing.

**Table 1:** Initial semen traits and post-thaw sperm quality.

	Boar 1	Boar 2	Boar 3	Boar 4	Boar 5
**Initial semen traits**					
Sperm concentration (×10^6^/mL)	254.1±21.1^b^ (230–296)	426.3±49.9^a^ (330–497)	247.1±11.5^b^ (224–261)	311.9±11.8^ab^ (288–324)	267.2±18.2^b^ (232–294)
Normal sperm morphology (%)	98.3±1.7 (95–100)	91.6±0.3 (91–92)	97±0.6 (96–98)	95±1.7 (92–98)	99±0.6 (98–100)
Total sperm motility (%)	81.1±3.2 (76–87)	78±1.2 (76–80)	83.3±0.7 (82–84)	73.7±3.2 (70–80)	82.7±1.8 (79–85)
Sperm viability (%)	89.7±0.9^x^ (88–91)	80.3±2.3^y^ (76–84)	85±2.5^xy^ (80–88)	84.5±3.5^xy .^(77.5–89)	83.3±1.2^xy^ (81–85)
**Post-thaw sperm assessments**					
Total sperm motility (%)	73.2±2.8^a^ (67–81)	47.3±2.6^c^ (40–56)	74.8±2.7^a .^(66–80)	67.3±3.1^ab^ (57–79)	56.8±1.2^bc^ (53–60)
Sperm viability (%)	73±1.8^a^ (69–77.4)	58±2.1^c^ (51.5–66)	75.2±1.8^a^ (70.8–81)	69.5±2.7^ab^ (58.1–76)	60.1±2.4^bc^ (53.5–69)
MDA generation (µmol/ 30×10^6^ cells)	7.6±1.1^b^ (2.7–11.9)	12.8±1.3^a^ (7.6–18.7)	11.6±0.7^a^ (8.3–15.1)	10.1±0.9^ab^ (5–13.9)	10.7±0.4^ab^ (8.7–13.3)

Superscripts indicate differences among boars: ^a–c^ p<0.01 and ^x–y^ p<0.05.

The initial semen traits and post-thaw sperm assessments of the native semen samples cryopreserved from the five boars. The data are the means ± SEMs of three ejaculates per boar, with ranges given between parentheses.

### Normalized post-thaw sperm motility and viability

**Figure 1:**
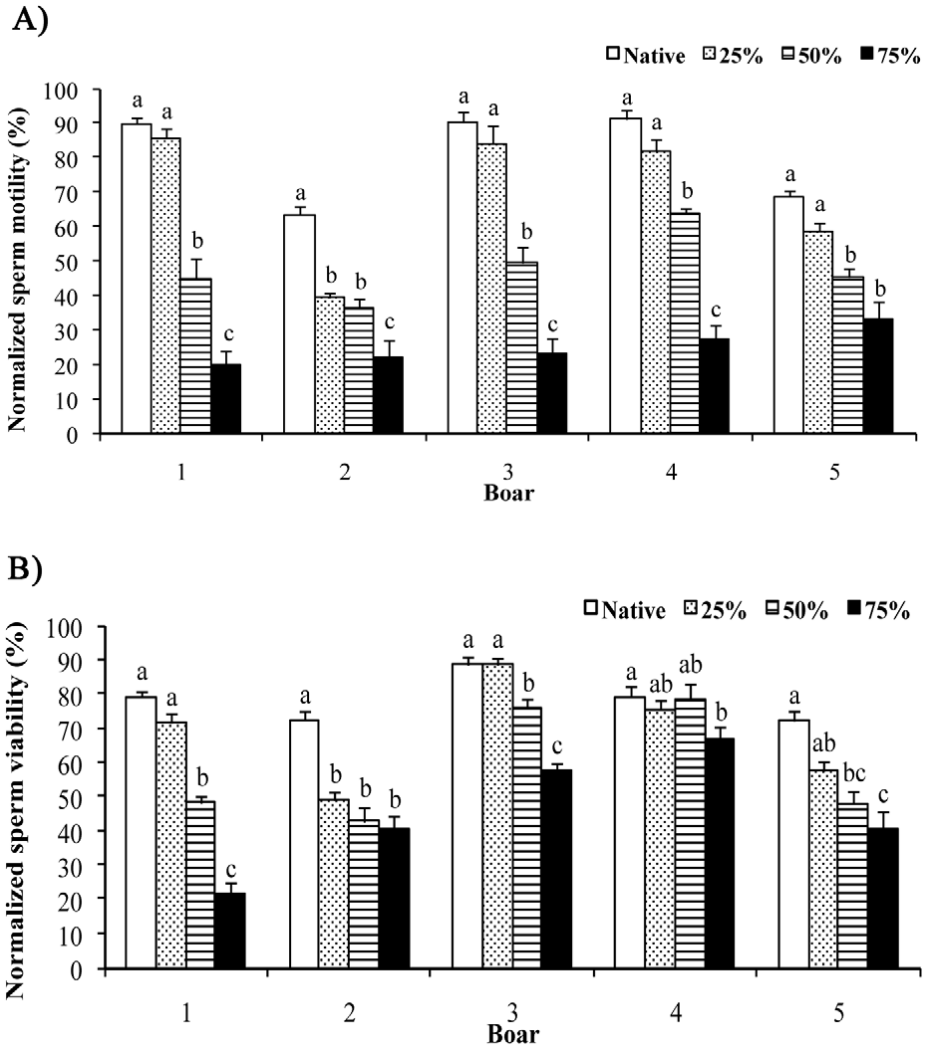
Effect of non-functional spermatozoa on post-thaw recovery of motile and viable spermatozoa. Post-thaw normalized sperm motility (a) and viability (b) of boar cryopreserved semen samples with different proportions of non-functional spermatozoa before freezing. The data are the means ± SEMs of 3 ejaculates per boar. Native is the original semen sample and 25–75% indicates the final proportion of non-functional spermatozoa in the handled semen samples. a–c indicate differences (p<0.05) among semen samples within the same boar.

Differences (p<0.01) were observed among boars and among semen samples within the same boar for normalized sperm motility and viability after thawing (Figure 1). The boar×semen sample interaction was also significant (p<0.01) for both sperm quality assessments. However, the five boars exhibited the same pattern, i.e., decreased normalized sperm motility and viability after thawing as the fraction of functional spermatozoa before freezing in the semen sample decreased. Therefore, the highest and the lowest normalized sperm motility and viability after thawing among the five boars were found in the semen samples with the highest and lowest proportions of viable spermatozoa before freezing, respectively.

### Membrane lipid peroxidation

**Figure 2:**
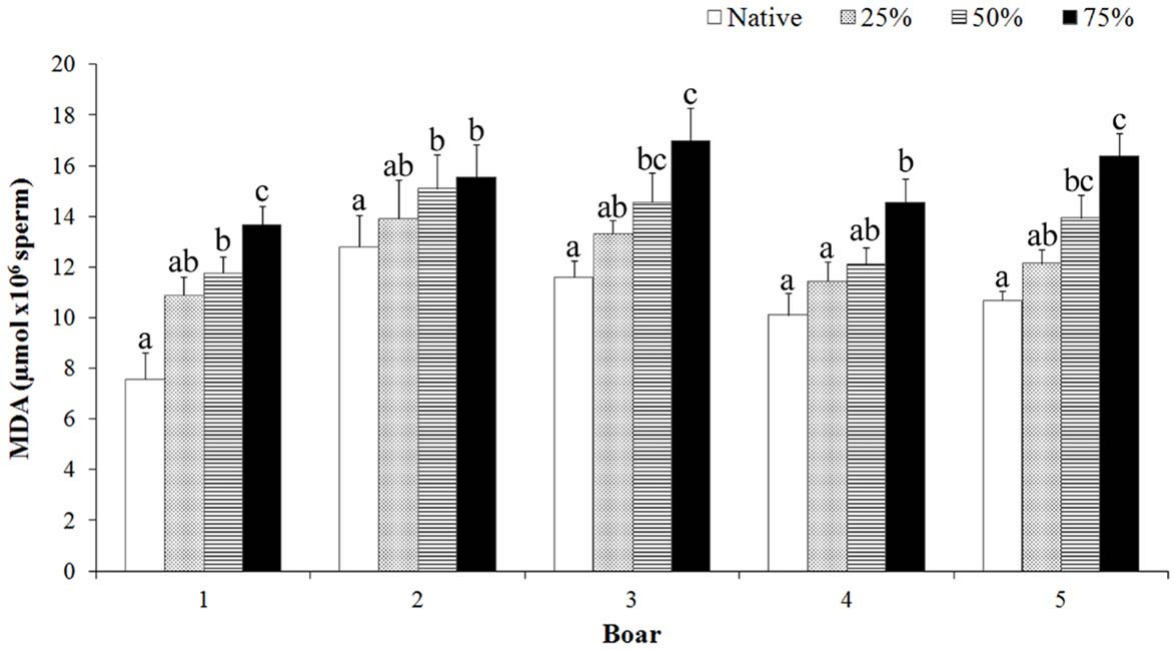
Effect of non-functional spermatozoa on post-thaw sperm membrane lipid peroxidation. Post-thaw malondialdehyde (MDA) generation in boar cryopreserved semen samples with different proportions of non-functional spermatozoa before freezing. The data are the means ± SEMs of 3 ejaculates per boar. Native is the original semen sample and 25–75% indicates the final proportion of non-functional spermatozoa in the handled semen samples. a–c indicate differences (p<0.05) among semen samples within the same boar.

The generation of MDA after thawing differed (p<0.01) among boars and among semen samples within the same boar (Figure 2). The boar x semen sample interaction was not significant. The generation of MDA in the five boars was inversely proportional to the proportion of functional spermatozoa in the semen samples before freezing: the semen samples with 75% non-functional cells and the native semen samples showed the highest and lowest MDA generation, respectively.

### Sperm functionality of post-thaw semen samples incubated under capacitating conditions

**Figure 3:**
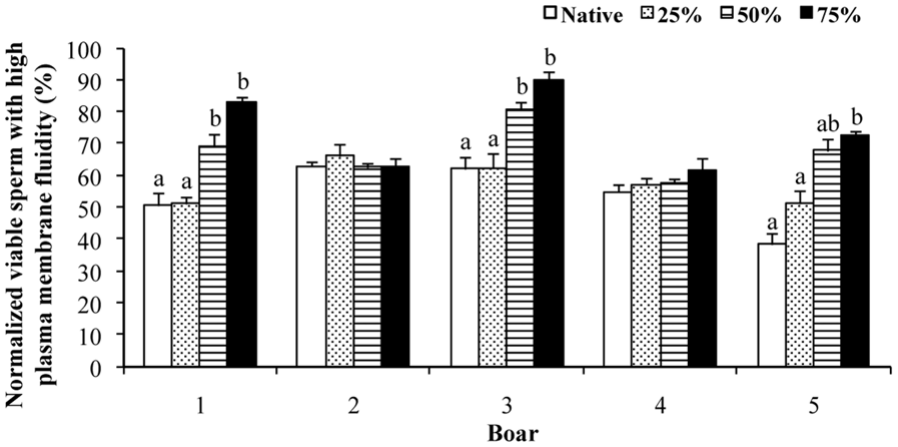
Effect of non-functional spermatozoa on post-thaw sperm plasma membrane fluidity. Plasma membrane fluidity (Yo-Pro–negative and M-540–positive) of thawed spermatozoa exposed to capacitation conditions from boar semen samples with different proportions of non-functional spermatozoa before freezing. The data are the means ± SEMs of 3 ejaculates per boar. Native is the original semen sample and 25–75% indicates the final proportion of non-functional spermatozoa in the handled semen samples. a,b indicate differences (p<0.05) among semen samples within the same boar.

The proportion of viable spermatozoa with high plasma membrane fluidity after thawing is shown in Figure 3. Differences (p<0.01) were observed among boars and among semen samples within each boar. The boar x semen sample interaction was significant (p<0.01). The pattern of boars 1, 3 and 5 differed from that of boars 2 and 4. In boars 1, 3 and 5, the proportion of viable spermatozoa with high plasma fluidity was higher in semen samples with 75 and 50% non-functional spermatozoa than in the native semen samples. In boars 2 and 4, the proportion of non-functional spermatozoa before freezing did not influence the normalized proportion of viable spermatozoa with high plasma membrane fluidity after thawing.

**Figure 4:**
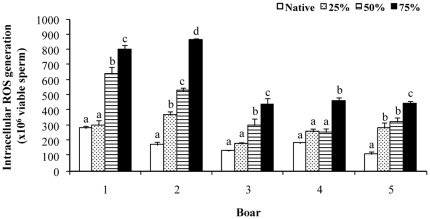
Effect of non-functional spermatozoa on post-thaw sperm intracellular reactive oxygen species generation. Intracellular generation of reactive oxygen species (ROS) in thawed viable spermatozoa exposed to capacitation conditions from boar semen samples with different proportions of non-functional spermatozoa prior freezing. The data are fluorescence units (means ± SEMs) of 3 ejaculates per boar. Native is the original semen sample and 25–75% indicates the final proportion of non-functional spermatozoa in the handled semen samples. a–d indicate differences (p<0.05) among semen samples within the same boar.

The intracellular ROS generation after thawing was expressed as FU/10^12^ live spermatozoa and is shown in Figure 4. Differences (p<0.01) were observed among boars and among semen samples within each boar. The boar x semen sample interaction was significant (p<0.01). However, the pattern of variation in the intracellular ROS generation among semen samples was similar in the five boars, with the lowest generation in the native semen samples and the highest in the semen samples with 75% non-functional spermatozoa before freezing.

## Discussion

To the best of our knowledge, this is the first experimental study to show the negative influence of non-functional spermatozoa in semen samples on the freezability of the functional spermatozoa. This adverse effect was concentration-dependent because the negative influence on the freezability of functional spermatozoa increased as the proportion of non-functional spermatozoa in the semen samples before freezing increased. This finding helps to explain the decrease in sperm survival and functionality during the freezing and thawing process, and it may provide insight for new techniques to enhance sperm cryosurvival, not only in pig ejaculates but also in the ejaculates of other mammalian species. In addition, the experimental model provides helpful insights into the biological significance of non-functional spermatozoa during the cryopreservation process. Therefore, the results could also be especially relevant for another mammalian species where the proportion of non-functional spermatozoa in the ejaculates is usually large, such in the human ejaculates, with percentages usually over 50% [27].

In the native semen samples, as expected, the cryopreservation process reduced the motility, progressive motility, and viability of the spermatozoa, irrespective of the semen donor. The magnitude of the reduction was boar-dependent, which was also expected. The individual variability in the capability of boar spermatozoa to withstand the freezing-thawing process [8], [28], [29] allows boars to be grouped as “good", “moderate" or “poor" sperm freezers on the post-thaw sperm quality [5], [30]. The presence of non-functional spermatozoa affected the cryosurvival of the functional spermatozoa, irrespective of the semen donor. As indicated by the boar x semen sample interaction, the magnitude of the effect differed among boars; that is, the spermatozoa from some boars were less susceptible than those from others. However, the magnitude of the effect was not related to the basal cryopreservation responses of the spermatozoa of the native semen samples, which suggests that non-functional spermatozoa affect the cryosurvival of functional spermatozoa independently of whether the boar is classified a “good" or “bad" sperm freezer. In contrast to the sperm freezer status, which seems to have a genetic basis [8], the inter-boar differences in the sensitivity of the functional spermatozoa to the negative effects of non-functional spermatozoa during cryopreservation could be related to differences in antioxidant SP components among individual boars or ejaculates [31].

It is also accepted that variation in the post-thaw sperm quality will occur among different ejaculates from the same boar [5] and that the optimization of the cryopreservation protocol can reduce the inter-boar and intra-boar variability in sperm cryosurvival [9], [32]. To optimize the cryopreservation protocols, the proportion of non-functional spermatozoa in the semen samples should be considered as a limiting factor for the selection of boar ejaculates for cryopreservation, not only because the non-functional sperm subpopulation minimizes the yield of the cryopreservation process but also because it actually reduces the freezability of the functional sperm subpopulation, as demonstrated in this study. Moreover, the cryosurviving sperm subpopulation of semen samples with a high proportion of non-functional spermatozoa before freezing is functionally different from that of samples with a low proportion of non-functional spermatozoa before freezing. These differences include a high generation of intracellular ROS and increased membrane fluidity.

The damaged sperm undergo structural changes among which include disarrangement of membranes with concomitant protein release, phospholipid scrambling, dissemination of the acrosomal content and lethal generation of ROS. Evidence from this experimental study suggests that oxidative stress may play an important role in the induction of lethal damage to functional spermatozoa during the cryopreservation. Oxidative stress results from an imbalance between the production of ROS and the antioxidant defense mechanisms. In this context, non-functional spermatozoa are an important source of ROS [33], [34]. Moreover, boar spermatozoa subjected to cryopreservation are particularly susceptible to oxidative attack, primarily because they contain large amounts of polyunsaturated fatty acids in their membranes [7] and because the SP, which is the main source of the antioxidant defense mechanism, is removed before freezing. Therefore, it is possible that the non-functional spermatozoa release high amounts of ROS in the medium that attack the membrane lipids of functional sperm, leading to the initiation of the LPO cascade. Lipid peroxidation damage can be quantified by measuring MDA, which is a stable by-product of LPO [35]. In the present study, significantly higher levels of MDA were measured after thawing in semen samples with a larger non-functional sperm subpopulation before freezing. LPO is results in the loss of sperm attributes, such as motility and membrane integrity, and finally in membrane degradation and sperm death. The loss of membrane integrity and the impairment of sperm motility are the major threats posed by the LPO cascade [23].

The non-functional spermatozoa also have detrimental effects on the functional of the cryosurviving spermatozoa, which exhibit both more endogenous ROS generation and increased membrane fluidity. In the present study, by using the H_2_O_2_-sensitive fluorescent probe H_2_DCFDA, we found that the levels of intracellular ROS, mainly H_2_O_2_, detected in the thawed viable spermatozoa exposed to capacitation conditions increased considerably in the semen samples with the highest proportion of non-functional spermatozoa before freezing (three to four times higher than those produced by thawed viable spermatozoa from native semen samples). H_2_O_2_ is the primary ROS generated by boar spermatozoa [23] and has been suggested to be the main ROS responsible for oxidative damage [22]. It is known that H_2_O_2_ is highly membrane-permeable and that upon release, it will attack the sperm membrane lipids, leading to the initiation of the LPO cascade [36]. Thawed viable spermatozoa exposed to capacitation conditions from semen samples with most non-functional spermatozoa before freezing also showed the highest percentage of spermatozoa with high plasma membrane fluidity. These functional changes indicate that cryosurviving spermatozoa from semen samples with high numbers of non-functional spermatozoa are more sensitive to premature capacitation, which could reduce their life span and compromise their ability to interact with the female reproductive tract [33].

Because the non-functional sperm subpopulation in the semen sample negatively influences the cryosurvival of the functional spermatozoa, the removal of non-functional spermatozoa from semen samples before freezing should be useful to improve the efficiency of cryopreservation. Several sperm selection techniques, such as migration-sedimentation, glass wool filtration and colloid centrifugation, permit the selection of higher-quality spermatozoa from the rest of the ejaculate [37]. However, these sperm selection techniques are not suitable for practical use in preparing sperm doses for animal AI because they are time-consuming and only work well with small volumes of semen. Recently, a more versatile colloid technique has been developed to work with large volumes of semen. The so-called single-layer centrifugation is effective in selecting the best sperm subpopulation from larger volumes of boar ejaculate [38] and could therefore be effective in removing the non-functional sperm subpopulation from boar semen samples before freezing, thereby improving the freezability of the boar ejaculates. This possibility requires further investigation.

In conclusion, the present study shows that non-functional spermatozoa in semen samples before freezing negatively influence the freezability of functional spermatozoa. This negative influence is evident not only by the reduced proportion of recovered functional spermatozoa after thawing but also by the altered functional of the sperm that survive the cryopreservation process.
